# Analysis of public perception and socio-demographic drivers of genetically modified organisms in Iran

**DOI:** 10.1080/21645698.2026.2668245

**Published:** 2026-05-07

**Authors:** Mohammad Ahmadabadi, Zahra Dehghanian, Rana Valizadeh Kamran, Maghsoud Pazhouhandeh, Ali Aryan

**Affiliations:** Department of Biotechnology, Azarbaijan Shahid Madani University, Tabriz, Iran

**Keywords:** Food security, genetically modified organisms (GMOs), Iran, logistic regression, public perception

## Abstract

The rapid growth in the world’s population poses a significant threat to food security, making genetically modified organisms (GMOs) an important potential solution. Alongside technological advancements, public awareness of GMOs – including their benefits and existing challenges – plays a crucial role in their acceptance and use within communities. This study aimed to assess opinions and the level of awareness of the Iranian public regarding GM products, the sources they consult for information on the topic, and their trust in these sources. A survey was conducted with 5,730 predominantly young, urban, and well-educated individuals from 16 provinces. The results revealed that 40% of respondents held positive attitudes toward GM crops, a figure higher than those reported in some social media analyses. Using multiple logistic regression, the study found that factors such as age, education, occupation, and residence significantly influenced attitudes toward GMOs, whereas gender did not. Although 42% of participants reported having limited knowledge about GMOs, most expressed greater trust in scientists compared to media sources and supported various applications of GMO technology. These findings, which focus on educated urban youth due to convenience sampling, underscore the importance of education and media in shaping perceptions of GM products in Iran.

## Introduction

Global food security is increasingly threatened by rapid population growth, climate change, water scarcity, and environmental degradation. In this context, genetic engineering and the development of genetically modified organisms (GMOs) have emerged as promising technologies that could enhance agricultural productivity and sustainability by addressing critical challenges.^1,2^ In recent decades, the development and use of genetically modified (GM) or transgenic plants have become significant topics of debate worldwide. GM technologies offer numerous advantages, including the potential to increase agricultural productivity, improve food security, and reduce environmental pollution by decreasing the use of chemical pesticides.^3,4^ However, concerns remain regarding the effects of GM products on human health, environmental safety, and ethical considerations, which have created barriers to their global acceptance.^5–7^ Several factors can influence public perception of GM products, including levels of scientific knowledge, cultural values, ethical concerns, and trust in mass media.^8–10^ Many individuals possess a limited understanding of genetic engineering and molecular breeding, often relying on media or social discussions for information.^9^ This lack of scientific knowledge frequently results in cautious attitudes or outright rejection of GM products.^11^

The adoption of new technologies is influenced by societal acceptance and public trust, in addition to their technical performance. When assessing the adoption of GM products in the field of biotechnology, it is essential to understand the knowledge and attitudes of different social groups.^9,12^ Iran has established a biosafety framework; however, strict regulatory controls govern the research and production of genetically modified goods.^13,14^ While academic and governmental institutions have conducted experimental research, commercial manufacturing remains limited due to legal restrictions, as well as cultural, religious, and public perception challenges. Iran’s regulatory structure is less uniform and is influenced by both scientific advisory organizations and Islamic law. This results in a more cautious approach compared to regions like the United States and the European Union.^13^ Regardless of the composition of genetically modified organisms, the Iranian Food and Drug Administration mandates that food items be labeled, demonstrating sensitivity to consumer concerns.

Public understanding and opinions regarding GMOs in Iran remain diverse and largely unexplored, according to empirical data. A study by Sheikhha et al.^15^ revealed significant differences in knowledge about GMOs between educated (79%) and non-educated (18%) individuals, with comparatively low perceived benefits among less-educated groups. More recent research^16^ has confirmed that mixed views – where both positive and negative perceptions coexist – are influenced by factors such as trust, health concerns, and social equity. Notably, a considerable portion of the population (39%) remains unaware of GMOs despite widespread exposure to discussions about them.^14^ Additionally, behavioral studies indicate that purchase intentions for GM products are significantly influenced by attitudes, perceived behavioral control, and subjective norms.^17^ Similar trends of insufficient understanding and generally cautious or unfavorable views have been observed in the Middle East, North Africa, and Turkey.^9^ Overall, these findings highlight that public acceptance of GM technology is shaped by levels of knowledge and education, risk-benefit perceptions, and sociocultural factors.

Due to challenges related to food security, such as population growth and climate change, Iran is increasingly importing genetically modified crops.^14,18^ This trend highlights the necessity of understanding public perceptions in order to inform evidence-based policies. Given Iran’s unique sociocultural, religious, and legal context, this understanding is particularly important. This study aims to address gaps in previous research by presenting the results of a comprehensive survey conducted across 16 regions in Iran, with a sample size of 5,730 participants. It primarily assesses Iranians’ opinions on GM products, their understanding of GMOs, the sources of information they rely on, and their trust in experts, utilizing logistic regression analysis. This research would be useful to address gaps in previous research, and will support evidence-based policymaking as well as communication strategies for improving public awareness and acceptance of GM technology in Iran.

## Materials and Methods

**Figure 1.**
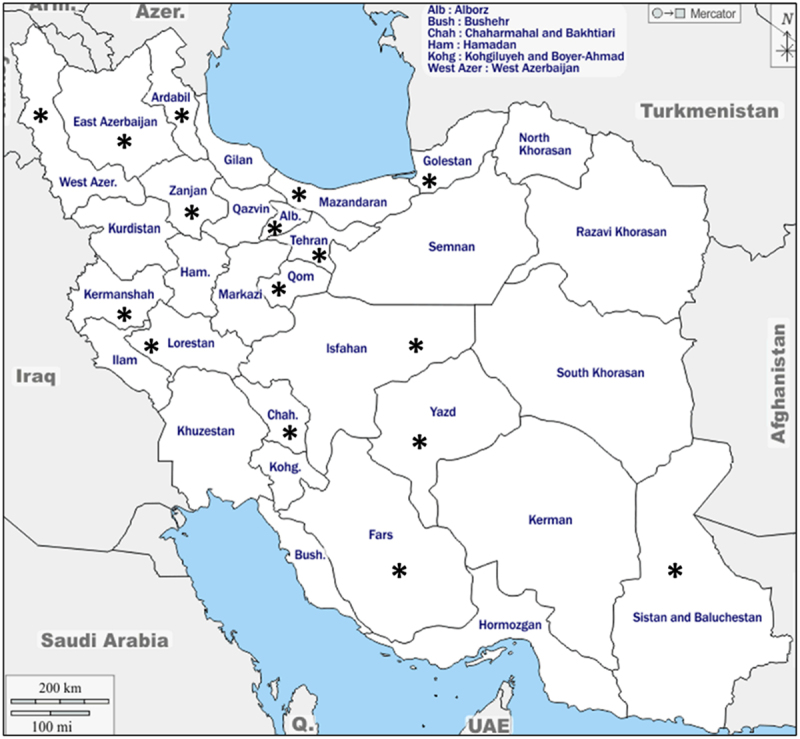
The map of Iran, displaying the 16 provinces evaluated, marked by asterisks.

This survey-based study was conducted from October 2022 to March 2025 to evaluate public opinion on GMOs and GM crops in Iran. A 20-question multiple-choice questionnaire (Table 1) was administered both online and in paper format to a diverse demographic of 5,730 participants across 16 provinces, including East Azarbaijan, West Azarbaijan, Ardabil, Zanjan, Qom, Chaharmahal and Bakhtiari, Tehran, Yazd, Golestan, Mazandaran, Lorestan, Fars, Kermanshah, Sistan and Baluchestan, Isfahan, and Alborz, to ensure geographic diversity (Figure 1). However, we acknowledge that the sample cannot be considered nationally representative. The large sample size (*n* = 5,730) was selected to provide sufficient statistical power and precise estimation of population parameters across various demographic subgroups and questionnaire items.

**Table 1.** Questionnaire items for the public survey on knowledge and opinions regarding genetically modified products.

KAP Domain	Question Code	Question
Knowledge	Q1	Do you think you have sufficient information about GMOs and GM products?
Knowledge	Q2	What is your primary source of information about GMOs?
Knowledge	Q3	To what extent have you obtained information about GMOs from the mass media?
Knowledge	Q4	In your opinion, are GM plants different from traditional ones?
Knowledge	Q5	Do you think animals in Iran are fed with GM feed?
Knowledge	Q6	Do you think GM food products are available in the Iranian market?
Knowledge	Q7	Do you think you have ever consumed GM food?
Knowledge	Q8	In your opinion, what type of information about GMOs could be the most important?
Knowledge	Q9	Each year, GM agricultural lands increase worldwide. In your opinion, what is the reason for this?
Attitude	Q10	What is your opinion about genetic modification and genetically modified organisms (GMOs)?
Attitude	Q11	In your opinion, what is the attitude of the mass media toward information about GMOs in Iran?
Attitude	Q12	How do you evaluate the role of mass media in informing the public about GMOs in Iran?
Attitude	Q13	Which source of information do you trust the most?
Attitude	Q14	Do you trust the opinions of Iranian scientists about GMOs?
Attitude	Q15	In your opinion, could GM products affect human health?
Attitude	Q16	What is your overall opinion about GMOs?
Attitude	Q17	How important is it to you to know whether a food product is GM?
Practice	Q18	For which purposes of GMO use in Iran do you agree?
Practice	Q19	If GM food products are labeled, how would this affect your purchasing decision?
Practice	Q20	If GM food products are available in the Iranian market, should they be labeled?

To evaluate the connections between awareness, perceptions, and behavioral intentions regarding GM crops, a questionnaire was developed based on the Knowledge-Attitude-Practice (KAP) framework. The questionnaire included nine questions (Q1–Q9) that focused on awareness, self-reported knowledge, and factual understanding – covering distinctions from conventional crops, market availability, and consumption history – in the knowledge domain. The attitude domain consisted of eight items (Q10–Q17) that assessed opinions, trust, perceived risks and advantages, media influence, and general perceptions. The practice domain featured three questions (Q18–Q20) related to behavioral intentions, including support for GM applications, labeling preferences, and purchasing decisions. Following traditional KAP methodology, each question was allocated to the most appropriate primary category, even though some items might overlap between domains. Participants were recruited through a convenience sampling method involving students from Azarbaijan Shahid Madani University and their referents, which may have influenced the demographic composition of the sample.

The demographic variables examined in this survey included gender, age group, education level, income, occupation, marital status, and place of residence. The survey revealed that 51.3% of the participants were male and 48.7% were female. A significant portion of respondents were young (57.8% aged 18–25), highly educated (64.4% holding a bachelor’s degree), and urban residents (74.9% living in a city center). The largest occupational group among the respondents was students, accounting for 49% of all participants. Detailed characteristics are summarized in Table 2.

**Table 2.** Demographic characteristics of participants in the survey on knowledge and awareness of genetically modified products.

Variable	Category	%
Age group	18–25	57.8
	26–35	19.5
	36–45	14.0
	46–55	7.3
	56 and above	1.4
Education level	Doctorate	4.0
	Master’s	10.1
	Bachelor’s	64.4
	Secondary	17.5
	Primary	4.0
Income Level (IRR) (million Iranian Rial)	Very low (<120.5)	30.4
	Relatively low (120.5–250)	32.3
	Moderate (250–500)	23.6
	High (>500)	13.8
Occupation	Physician	2.8
	Faculty member	1.6
	Employee	16.9
	Worker	1.4
	Farmer	1.2
	Self-employed	9.1
	Student	49.0
	Housewife	8.9
	Other	8.9
Marital status	Single	58.5
	Married	40.0
	Other	1.6
Place of residence	City center	74.9
	Suburb	17.3
	Village	7.9

Note: Income levels are approximate monthly ranges in Iranian Rials (IRR), based on 2025 economic data and adjusted for inflation (as per the Central Bank of Iran’s 2025 estimate).

To guide the statistical analysis, hypotheses were formulated within the KAP framework, based on prior research from Iran and the Middle East, North Africa, and Turkey (MENAT) region. We hypothesized that (H1) younger, more educated, and urban respondents would exhibit more positive attitudes toward GM crops; (H2) no significant gender differences would be observed in attitudes; (H3) higher levels of self-reported knowledge would be associated with more favorable attitudes and greater support for GM applications; (H4) trust in scientists, compared to mass media, would be higher and positively associated with GMO acceptance; and (H5) socio-demographic factors – particularly age, education, occupation, and residence – would significantly influence knowledge, attitudes, and practices, whereas income and marital status would have weaker effects. These hypotheses are consistent with previous findings linking education, awareness, and institutional trust to public acceptance of GM technologies.^9,14,16^

Demographic characteristics of the respondents were summarized using descriptive statistics, including frequency and percentage. To examine the effect of demographic variables on responses to the questionnaire items (Table 1), multiple logistic regression (MLR) was employed, where each survey item was treated as a dependent variable, with appropriately categorized response options. Demographic variables (such as age, education, income, etc.) were included in the model as independent variables. Additionally, to enhance the analysis and provide clearer findings, cross-tabulations (crosstabs) and column percentages were calculated to display patterns of response choices among different demographic groups. Model performance was evaluated using likelihood-based statistics Nagelkerke’s R^2^, and results are reported as odds ratios (OR) with 95% confidence intervals (CI). To complement the regression analysis, non-parametric methods were applied to assess differences across groups for ordinal and categorical variables. The Kruskal – Wallis H and Jonckheere – Terpstra (JT) tests were used for multi-group comparisons, with effect sizes estimated using eta-squared (η^2^). When significant differences were detected, Bonferroni-adjusted post hoc tests were conducted for multiple comparisons. All statistical tests were two-tailed, with significance set at *p* < .05. Analyses were performed using IBM SPSS Statistics version 24, allowing for a deeper understanding of the socio-demographic factors influencing public perception.

## Results

**Figure 2.**
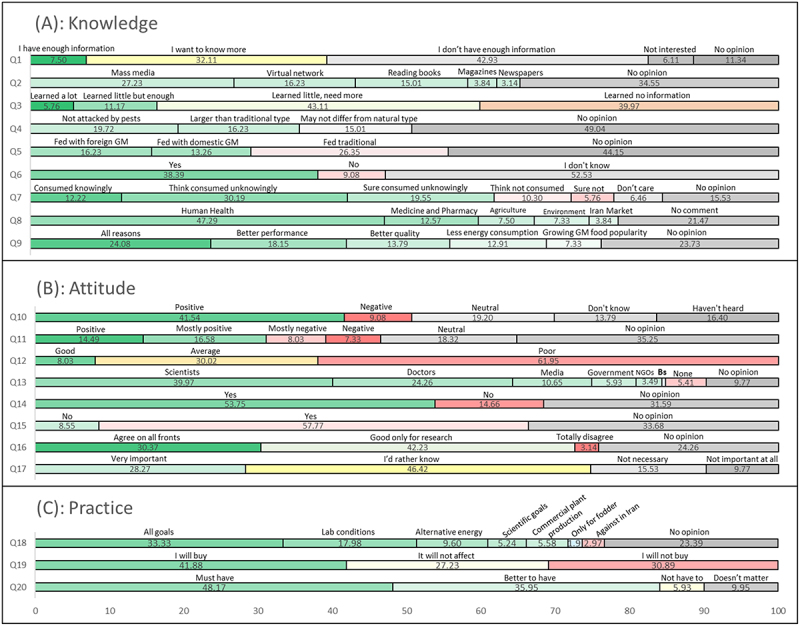
Percentage distribution of participant responses (*n* = 5,730) to the questionnaire items organized according to the knowledge-Attitude-practice (KAP) model. The figure shows participants’ overall (A): knowledge and awareness, (B): attitude and perception, and (C): behavioral intentions, regarding GM crops. (Bs: Businessman. Consistent color scheme: green or light green: Positive/favorable/knowledgeable responses; red or orange-red: Negative/concerned/limited responses; yellow: “want to know more” or “I’d rather know”; Gray: neutral / “no opinion” / “Don’t know”).

The results obtained from the completed survey questionnaires are presented as percentages in Figure 2. A total of 5,730 respondents participated in the study. The multinomial logistic regression models demonstrated an overall acceptable fit to the data. Descriptive analysis based on the KAP framework revealed diverse levels of knowledge, attitudes, and behaviors regarding GM crops.

### Knowledge Domain (Questions 1–9)

Several socio-demographic factors were significantly correlated with respondents’ knowledge (Table 3). Education level emerged as a significant predictor of awareness (Q1; OR = 5.05, 95% CI: 0.88–28.89) based on MLR. Knowledge variation also showed a strong correlation with age group (Q2; OR = 43.47, 95% CI: 1.40–100). Additionally, responses were closely linked to income level (Q3; OR = 3.33, 95% CI: 1.05–11.04). Non-parametric analysis supported these findings. Jonckheere-Terpstra tests indicated a strong positive trend between education level and knowledge (Z = −3.261, *p* = .001), while Kruskal-Wallis tests identified significant differences across age, education, and profession groups (*p* < .05). However, minor effect sizes (η^2^ < 0.06) suggest limited practical importance.

**Table 3.** Summary of multinomial logistic regression and non-parametric test results for questionnaire items.

QC	Gn	AG	EL	In	Oc	MS	RP	OR (95% CI) – MP	Key Non-Parametric Results	
									Kruskal-Wallis	Jonckheere–Terpstra
Q1	—	* p = .011	* p = .001	—	* p = .026	—	—	EL:5.056 (0.885–28.89)	AG: H = 11.323, *p* = .023, η^2^ = 0.0128 EL: H = 20.01, *p* = .001, η^2^ = 0.028 Oc: H = 28.150, *p* = .001, η^2^ = 0.036	AG: (Z = 2.267, *p* = .022) EL: (Z = −3.261, *p* = .001)
Q2	—	* p < .001	—	* p = .001	—	—	* p = .001	AG: 43.47 (1.4–100)	AG: H = 13.292, *p* = .01, η^2^ = 0.0163 In: H = 15.447, *p* = .001, η^2^ = 0.022 Oc: H = 37.135, *p* = .001, η^2^ = 0.060	AG: (Z = 2.297, *p* = .001). In: (Z = 3.845, *p* = .001)
Q3	—	—	—	* p = .007	—	—	—	I: 3.331 (1.05–11.04)	In: H = 15.4, *p* = .002, η^2^ = 0.022	In: (Z = 3.2275, *p* = .001)
Q4	—	—	—	—	—	—	—	—	—	—
Q5	—	—	—	—	—	—	—	—	—	—
Q6	—	* p = .015	—	—	—	—	—	—	AG: H = 9.027, *p* = .006, η^2^ = 0.0088	—
Q7	—	—	* p = .023	—	* p = .014	* p = .01	—	EL: 36.97 (2.911–469.57)	EL: H = 9.903, *p* = .042, η^2^ = 0.059 Oc: H = 36.617, *p* = .001, η^2^ = 0.050 MS: H = 11.352, *p* = .003, η^2^ = 0.017	EL: (Z = −3.251, *p* = .002)
Q8	—	—	—	—	—	* p = .008	—	—	—	—
Q9	—	—	—	—	—	—	* p = .004	RP: 8.376 (0.998–70.30)	RP: H = 10.606, *p* = .005, η^2^ = 0.015	—
Q10	—	* p = .008	* p = .003	—	—	—	* p = .001	EL: 18.69 (2.86–121.9)	EL: H = 37.397, *p* = .001, η^2^ = 0.016 RP: H = 37.135, *p* = .001, η^2^ = 0.06	EL: (Z = −5.74, *p* = .001)
Q11	—	—	—	—	—	—	—	—	—	—
Q12	—	—	—	—	—	—	* p = .009	—	RP: H = 7.836, *p* = .02, η^2^ = 0.013	—
Q13	—	* p = .001	—	—	—	—	—	AG: 19.755 (2.41–161.93)	—	—
Q14	—	—	—	—	—	—	—	—	—	—
Q15	—	—	—	—	—	—	—	—	—	—
Q16	—	—	* p = .001	* p = .001	* p = .001	* p = .001	—	EL:8.712 (1.64–46.17)	EL: H = 14.151, *p* = .007, η^2^ = 0.02 In: H = 8.596, *p* = .035, η^2^ = 0.009 MS: H = 9.565, *p* = .008, η^2^ = 0.013	In: (Z = 2.818, *p* = .004)
Q17	—	* p = .016	—	—	* p = .001	—	—	—	AG: H = 15.006, *p* = .005, η^2^ = 0.0193 Oc: H = 30.548, *p* = .001, η^2^ = 0.040	AG: (Z = 3.291, *p* = .001)
Q18	—	—	—	—	—	—	—	—	—	—
Q19	—	—	—	—	—	—	—	—	—	—
Q20	—	—	—	—	—	—	—	—	—	—

Notes: QC: Question Code, Gn: Gender, AG: Age Group, EL: Education Level, In: Income, Oc: Occupation, MS: Marital Status, RP: Residence Place, MP: Main Predictor. Asterisks (*) indicate significant effects (*p* < .05) in multiple logistic regression. Main predictor was defined as the demographic variable with the strongest statistically significant association (*p* < .05) based on multinomial logistic regression analysis. Odds ratios (OR). Non-parametric tests included Kruskal–Wallis (KW) and Jonckheere–Terpstra (JT) tests. η^2^ and r: Effect sizes for KW, Z: standardized test statistics for JT.

Response distributions indicated a generally low level of awareness (Figure 2A): 42% of participants reported knowing very little about GMOs (Q1), while most expressed a desire to learn more. The primary source of information was the mass media (Q2), although the depth of this information was limited (Q3). Despite the fact that GM food is imported into Iran,^14,18^ over 60% of respondents were unaware that GM food was available domestically (Q6). Additionally, there was uncertainty regarding the use of GM feed in animals (Q5) and prior consumption of GM foods (Q7). Perceptions of the global spread of GM agriculture varied widely (Q9), and many respondents were unclear about the differences between GM and traditional crops (Q4).

### Attitude Domain (Questions 10–17)

Stronger and more reliable correlations with socio-demographic factors were observed in the attitude domain (see Table 3). Higher education levels were associated with more positive opinions about GM crops. This is evident in questions Q10 (OR = 18.69, 95% CI: 2.86–121.9) and Q16 (OR = 8.71, 95% CI: 1.64–46.17), where education level emerged as the most significant predictor. Age group also played an important role, particularly in Q13 (OR = 19.75, 95% CI: 2.41–161.93). Non-parametric studies supported these findings. The Jonckheere – Terpstra tests indicated a consistent positive trend with increasing education levels (Z = −5.74, *p* = .001), while the Kruskal – Wallis tests revealed significant differences across age, income, and education groups (*p* < .01). The effect sizes showed modest practical significance, ranging from small to moderate (η^2^ = 0.01–0.06).

Overall, opinions on GM crops were divided. Consistent with previous research,^16^ nearly 40% of respondents held a favorable view of genetic modification (Q10; Figure 2B). In terms of application, 42% preferred the use of GM products primarily for research purposes, while 29% supported their use in all sectors (Q16; see Figure 2B). Younger respondents tended to be significantly more optimistic. Scientists were identified as the most reliable source of information, with 70% of participants expressing this view in Q13, far outperforming government organizations and the media. However, more than 40% of participants expressed concerns about potential health risks (Q15), and many emphasized the importance of labeling genetically modified products (Q17). In general, respondents felt that the media’s role in educating the public about genetically modified crops was inadequate (Q11–Q12).

### Practice Domain (Questions 18–20)

In comparison to the knowledge and attitude domains, the practice domain showed fewer significant correlations with socio-demographic characteristics (Table 3). Most factors did not display statistically significant correlations with behavioral responses, and questions Q18 and Q19 did not have any meaningful predictors. Although there was a considerable change in Q20, these effects were less consistent. This suggests that knowledge and attitudes may play a more important role in mediating behavioral intentions than socio-demographic traits.

Concerning stated practices, 29% of respondents supported the use of GM crops in all sectors, while 42% supported their use solely for scientific or research purposes (Q18; Figure 2C). About 36% of respondents believed that GM product labeling was desirable, and many indicated that labeling would influence their purchasing decisions (Q19). Accordingly, 48% of participants stated that GM food items should be labeled if available in the market (Q20).

#### Consistency and Robustness

The robustness of the observed relationships was supported by the results of non-parametric studies, which generally aligned with those from the multinomial logistic regression. The primary findings remained statistically significant even after applying the Bonferroni adjustment (α = 0.0025) to account for multiple comparisons. The knowledge domain displayed satisfactory internal consistency, with a Cronbach’s α of 0.715. In contrast, the attitude and practice domains showed lower internal consistency (α = 0.579 and α = 0.249, respectively), indicating that the results from these areas should be interpreted with caution.

## Discussion

This study utilizes the Knowledge-Attitude-Practice (KAP) framework to systematically analyze the transition from awareness to behavioral intentions regarding genetically modified crops among the Iranian public. It represents a comprehensive evaluation and employs a 20-item questionnaire, which is organized into three categories: knowledge (Q1–Q9), attitude (Q10–Q17), and practice (Q18–Q20). This structured approach aligns with similar research conducted in Iran,^14,16^ China,^19^ Lebanon,^20^ and the MENAT region.^9^ Key findings from the study also support Risk Perception Theory, emphasizing the roles of perceived risk, uncertainty, and institutional trust.^21^ The results from MLR analysis suggest that socio-demographic variables play a significant role in shaping public perceptions of GMOs. Notable results include significant support for mandatory labeling of GM products (48.2%, Q20; Figure 2C) and ongoing concerns about potential health impacts (57.8%, Q15; Figure 2B). The integration of these frameworks underscores the importance of open communication and trust-building strategies, suggesting that increased knowledge does not necessarily lead to a reduction in perceived risks.

Public attitudes toward GM foods exhibit both continuity and progressive change when compared to previous research conducted in Iran. According to Sheikhha et al.,^15^ there are significant gaps in awareness and a limited perception of the advantages of these foods. Recent studies have confirmed that knowledge gaps and conflicting opinions persist, influenced by social factors, trust, and health-related concerns.^14,16^ Behavioral research further demonstrates the impact of subjective standards and perceived control on consumer intentions.^17^ Similar cautious attitudes and insufficient knowledge have also been observed in the MENAT region.^9^ While knowledge gaps and health concerns remain common, our findings with a large sample (*n* = 5,730) indicate some progress, particularly among younger, more educated, and urban respondents.

Our results showed that nearly 40% of respondents hold favorable opinions about GM crops, indicating that Iranians exhibit relatively favorable attitudes toward GMOs compared to global trends.^22^ However, a recent study claims an over 80% positive attitude toward GE foods in China.^19^ Notably, in our study, the support was stronger for research purposes than for large-scale commercial use (Q10, Q16, Q20; Figure 2). The primary factors influencing acceptance levels are age and education; these levels appear to be higher than those reported across the MENAT region.^9^ Generally, younger, more educated individuals showed more favorable views toward GM products. While conflicting evidence exists (Pew Research Center: https://www.pewresearch.org/?p=10182), these finding align with international research that links education and perceived benefits with acceptance.^20,23^ Additionally, studies suggest that younger generations may perceive biotechnology as a solution to food security challenges.^24,25^ Unlike some international findings that report gender differences – with men generally more positive than women ^26–28^—our study found no significant gender effects across all 20 questions, possibly due to the relatively high level of educational attainment among women in Iran,^29^ which has been shown to have positive correlation with knowledge levels.^20^ Similar trends were observed in Japan, where higher awareness contributed to increased acceptance of biotechnology.^30^

Trust in information sources was found to be a key factor shaping attitudes. Consistent with previous survey results,^31^ most respondents indicated greater trust in information from scientists than in media announcements (Q13; Figure 2B). However, despite this trust, the majority of participants rely on the media as their primary source of information, often receiving limited or insufficient content (Q2; Figure 2A). In a recent study, training programs and seminars has been reported to be the most favored method of information dissemination in Tanzania.^32^ These highlight the need for improved scientific communication, which should include fair media coverage and expert-led educational programs.

The gap between feeling informed and a lack of knowledge highlights an important issue in public communication. Previous studies show that a substantial number of Iranians are unaware of GM crops and express doubts about their availability in local markets.^14,15^ Consistent with findings from Akbari et al.,^16^ concerns about potential health risks were prevalent, with approximately 58% of respondents believing that GM products could be harmful (Q15; Figure 2B). Despite a large body of scientific evidence confirming the safety of GM products,^3,33–35^ these concerns persist. Biased or insufficient information, {as reflected in our results (Q1; Figure 1A) as well as previous reports from Tehran/Iran ^14^}, can reinforce misconceptions, underscoring the importance of evidence-based public communication. In addition, similar to a previous study in China,^36^ our findings indicated that urban residents tend to have more positive views on GM crops. This suggests the need for targeted outreach programs in rural areas, which may be harder to reach. Additionally, consistent with the KAP framework, higher levels of knowledge about GM applications were associated with more favorable views and support, while acceptance was notably influenced by trust in scientists.

Although not specifically examined in this study, cultural and religious influences likely impact attitudes toward GM products. In Iran, for example, GM food are generally considered acceptable under Islamic beliefs, provided they comply with halal regulations.^37^ Given the strong trust in scientific authority, collaboration between scientific and religious organizations could help address public concerns and enhance acceptance.

The importance of transparency is further illustrated by behavioral responses. Despite health concerns, a significant percentage (42%) of respondents indicated they would be willing to consume GM foods if they were properly labeled (Q19; Figure 2C), and more than half of them supported mandatory labeling, as documented previously.^14,35,38^ Transparency through labeling – already in place in Iran but underutilized ^14,38^—could enhance trust,^36^ particularly as many believe they have already consumed GM products without knowing (Q7, Figure 1A), which aligns with the importation of GM crops like corn and soybeans. However, differences in labeling knowledge (Q17; Figure 2B) reveal persistent educational gaps. These findings underscore the need for public education and clear labeling regulations to promote informed consumer choices. Furthermore, public perceptions of genetically modified crops are often influenced by lobbying organizations and a media environment that tends to emphasize criticism.^39^ As a result, skepticism resonates more with a generation raised to distrust large corporations and established institutions.

Lastly, a few limitations should be considered. Convenience sampling may limit generalizability, as it tends to overrepresent young, educated, urban respondents. Future research should utilize stratified sampling and consider external factors to enhance validity. Additionally, the extended data collection period from 2022 to 2025 may introduce temporal effects.^40^ It is also important to acknowledge potential response and social desirability biases, as well as the relatively poor internal consistency in the attitude and practice domains. Comparisons with international data are provided for contextual purposes rather than direct methodological equivalence.

## Conclusion

This survey indicates that there is still a significant knowledge gap among the Iranian public, despite generally positive opinions regarding GM technology. The most reliable predictors of knowledge and attitudes were found to be age and education level. Higher education is linked to greater awareness and more favorable opinions, although the effect sizes were typically small to moderate. Additionally, socio-demographic characteristics showed little correlation with the practical application of GM technology. These results highlight a concerning disparity between behavioral intentions, attitudes, and knowledge, underscoring the need for proactive and planned public communication. Future communication strategies should be led by scientists, who enjoy a high level of public trust. These efforts should focus on translating complex biotechnology concepts into easily understandable information rather than adopting a defensive stance against opposing views. Policymakers are encouraged to implement evidence-based policies, such as transparent labeling that explains the safety and benefits of genetically modified foods. The media should also shift from inadequate coverage to fair, expert-driven discussions. To address ongoing issues and close existing knowledge gaps, consistent funding for independent research and targeted educational initiatives will be essential. Overall, this study provides a comprehensive understanding of Iranian public opinions on genetically modified crops and offers practical suggestions for improving public acceptance and informing policy development.
